# Squamous Eccrine Ductal Carcinoma: A Comprehensive Review of the Clinicopathologic Features and Management in 27 Cases

**DOI:** 10.7759/cureus.87037

**Published:** 2025-06-30

**Authors:** Santiago Gudino-Rosales, Cullen M Lilley, Gregory Gates, Diana Kneiber

**Affiliations:** 1 Department of Dermatology, University of California Los Angeles, Los Angeles, USA; 2 Department of Pathology and Laboratory Medicine, University of California Los Angeles, Los Angeles, USA

**Keywords:** cutaneous neoplasm, eccrine carcinoma, rare skin lesion, skin cancer, squamoid eccrine ductal carcinoma, squamous cell carcinoma

## Abstract

Squamoid eccrine ductal carcinoma (SEDC) is a rare cutaneous malignancy that demonstrates both squamous and eccrine differentiation. With a limited number of published cases in the literature, SEDC remains a diagnostic challenge. This retrospective chart review reports on 27 cases of SEDC identified at our institution and summarizes their clinical and histopathologic characteristics. Clinically, these tumors demonstrated a predilection for sun-exposed areas, favoring the head and neck (59.2%, n=16) of older individuals (median age 74 years). They presented as a papule, nodule, or plaque (48.1%, n=13) and were less than 2 cm in size. Despite surgical treatment with either conventional wide local excision or Mohs micrographic surgery, they had a local recurrence rate of 18.5% (n=5). One patient had metastatic disease with local recurrence, regional lymph node involvement, and distant skin metastasis, which led to the patient’s death. Given that SEDC histologically exhibits both squamous and eccrine differentiation and immunophenotypically expresses markers of both eccrine/adnexal and squamous origin, this carcinoma poses a diagnostic challenge, as superficial biopsies may be indistinguishable from squamous cell carcinoma. This study emphasizes the importance of recognizing this tumor due to its recurrence risk and potential for metastasis and mortality.

## Introduction

Squamoid eccrine ductal carcinoma (SEDC) is a rare and underrecognized cutaneous carcinoma. It is believed to arise from eccrine glands and predominantly presents in sun-exposed areas of the head and neck as either a nodule or plaque [1]. Histologically, it demonstrates both squamous and adnexal ductal differentiation, which makes diagnosis particularly challenging, as a superficial biopsy can be incorrectly identified as a squamous cell carcinoma [2]. Although first described in 1991, SEDC continues to be underreported, with only 127 cases published in the literature [2-11]. Consequently, its exact incidence, disease progression, and optimal treatment approach remain unclear. Despite its rarity, SEDC is clinically significant given its potential for recurrence and metastasis [3]. This study aimed to contribute to the extant literature by describing the clinicopathologic features, treatment outcomes, and recurrence patterns of 27 cases of SEDC. In doing so, the authors aimed to provide further insights into this rare skin cancer, which may inform diagnosis and guide clinical decision-making.

## Materials and methods

Patient information was deidentified and data were gathered after approval from the institutional review board. Data were gathered via retrospective chart review of the University of California, Los Angeles (UCLA) Health medical records to identify cases of squamoid eccrine ductal carcinoma from January 1, 1990, to September 30, 2024. To identify potential cases, an internal case search function built into our Epic Systems' Beaker Laboratory Information System database was utilized. The search we executed was a natural language search. Initial screening located 35 medical records, of which the authors identified 27 cases with pathology reports consistent with squamoid eccrine ductal carcinoma. We excluded any duplicates and cases with pathology reports that were not consistent with SEDC. Patient records were examined for various clinical data, including patients’ age at diagnosis, ethnicity, skin phototype, dermatologic history, personal and family history of cancer, clinical descriptions, and clinical course of SEDC lesions. Lesion management was reviewed through surgical reports of either wide local excisions or Mohs micrographic surgery and documentation of adjuvant radiation or chemotherapy. Clinical follow-up data were obtained and averaged in months from the initial surgical treatment of the lesion to the most recent clinic visit to oncology, dermatology, primary care, or surgery for routine clinical surveillance. Nineteen cases were identified as pathology consults with limited histopathologic data, and eight were available for secondary histologic examination with H&E staining and CEA immunohistochemistry by a board-certified dermatopathologist.

## Results

Clinical data

The median age at diagnosis was 74 years, with a range from 55 to 94 years. Nineteen cases had ethnicity data, with all of them identifying as either White or Caucasian. Fitzpatrick data were available for 15 cases with skin types ranging as follows: I-II (53.3%, n=8), II (20%, n=3), and II-III (26.7%, n=4). A total of 70.4% (n=19) of cases had documented histories of skin cancer with squamous cell carcinoma being highest (55.6%, n=15), followed by basal cell carcinoma (40.7%, n=11), melanoma (18.5%, n=5), and SCC vs. syringoid carcinoma (3.7%, n=1). A family history of skin cancer was reported in 18.5% (n=5) of cases, and 29.6% (n=8) had histories of smoking tobacco. Nine cases also had histories of non-skin cancers as follows: breast ductal carcinoma (two cases), prostate carcinoma (two), breast adenocarcinoma and cervical cancer (one), unspecified thyroid cancer (one), rectal adenocarcinoma (one), myelodysplastic syndrome (one), and chronic lymphocytic leukemia, mucoepidermoid carcinoma, and prostate carcinoma (one).

**Figure 1:**
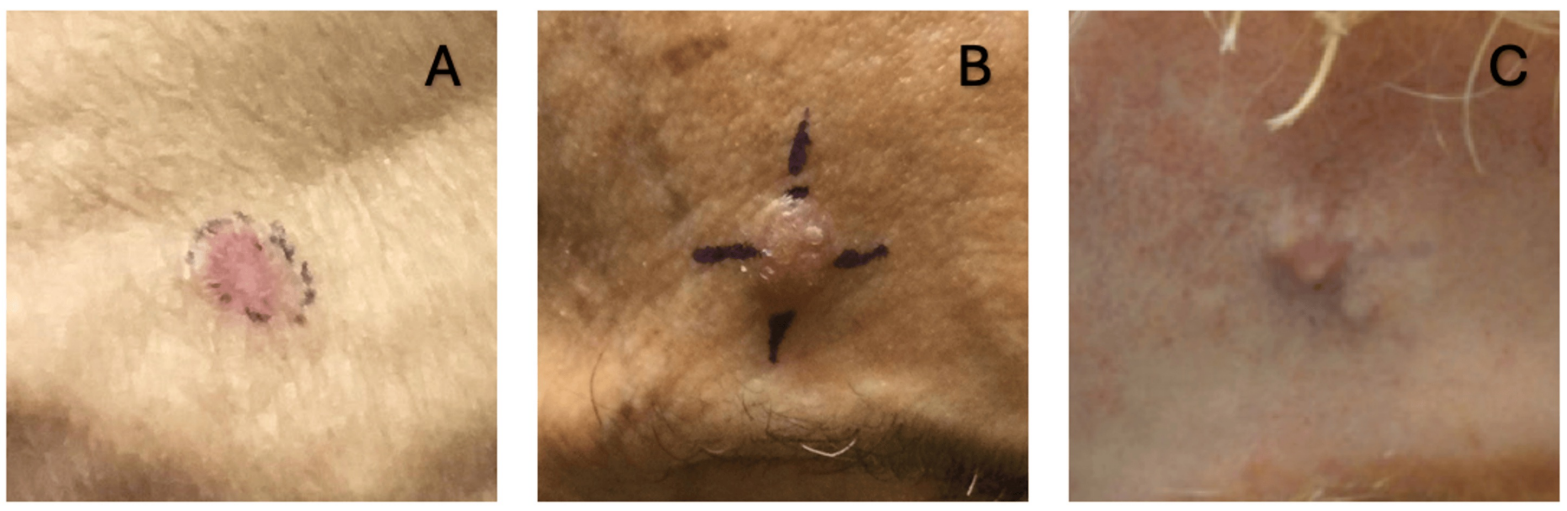
Clinical images of three patients with SEDC. (A) Well-defined pink plaque on the shoulder. (B) Eroded papule with crusting on the forehead. (C) Mobile mass on the forehead. SEDC: squamoid eccrine ductal carcinoma

Head and neck were the most common locations (59.2%, n=16) with less common areas being the trunk (26%, n=7) and extremities (14.8%, n=4). Clinical descriptions of initial lesions varied greatly in documented morphologies with nodules and papules being the highest reported descriptions (37%, n=10), followed by plaque (11.1%, n=3), ulcer (3.7%, n=1), and mass (3.7%, n=1) (Figure 1, panels A-C). Symptoms similarly varied in presentation with firmness (11.1%, n=3), pain (11.1%, n=3), bleeding (7.4%, n=2), erythema (7.4%, n=2), and growth (7.4%, n=2) being common symptoms. Less commonly reported symptoms included pruritus (3.7%, n=1), scaling (3.7%, n=1), crusting (3.7%, n=1), inflammation (3.7%, n=1), and burning (3.7%, n=1).

A total of 51.9% (n=14) cases had previously been seen by a dermatologist. Dermatology was the specialty service that performed the majority of biopsies (92.6%, n=25), with plastic surgery completing one scalp biopsy and general surgery performing a re-excisional biopsy. Most biopsies (88.9%, n=24) were performed on the same clinical visit as the initial concern regarding the lesion, with one biopsy completed on the second visit after determining the specialty service that would perform it.

Treatment analysis

Of the 27 patients, 17 cases had information regarding their treatments, with nine that underwent wide local excision (WLE), seven that were managed with Mohs micrographic surgery (MMS), and one that received both WLE and MMS.

Of the nine cases managed with WLE, surgical reports were available for seven (Table 1). Patients one and four were cases of recurrent SEDC lesions that had been previously managed with MMS, for which initial MMS data were unavailable. Ultimately, after repeat surgery with WLE, both demonstrated clearance at respective one-year and 11-year follow-ups, with patient one having received additional adjuvant radiotherapy. Four patients (one, four, five, and six) required repeat excision due to positive margins on initial WLE, performed within three days to three months of the initial excision. Though patient three was the only case to receive chemotherapy specifically for SEDC management, patients four and five also received chemotherapy (ipilimumab/nivolumab and ipilimumab, respectively) after their excisions for concurrent melanoma management. Only two patients (two and seven) achieved clearance after a single excision without needing any additional excisions, adjuvant radiation, or chemotherapy. The clinical course of patient three is discussed in the recurrence and metastasis analysis sections.

**Table 1:** Details of wide local excisions. *Treatment for recurrent lesions previously managed with MMS. **Received chemotherapy for management of concurrent melanoma. MMS: Mohs micrographic surgery

Patient	Location	Treatment specialty	Pre-operative size (cm)	Margins (mm)	Post-operative size (cm)	Adjuvant radiation	Chemotherapy	Follow-up (months)
1*	Scalp	Head and neck	1x0.6x0.3	10	6x6	No	No	-
	Scalp re-excision	Head and neck	6x6	10	12x6	Yes	No	22
2	Scalp	Head and neck	1.5x1.5	10	4x3	No	No	-
3	Shoulder	General surgery	5	25	10x5	No	No	-
	Shoulder re-excision	General surgery	2.1x1.2	-	4x2x1.1	Yes	Yes	39
	Forehead	General surgery	1.4x1.1	-	1.8x0.5x0.4	Yes	Yes	39
4*	Scalp	Surgical oncology	-	10	4x4	No	Yes**	-
	Scalp re-excision	Surgical oncology	4x4	20	10x5	No	Yes**	134
5	Forehead	Surgical oncology	1.5x2	-	3x3	No	Yes**	-
	Forehead re-excision	Plastic surgery	3x3	40	-	No	Yes**	16
6	Neck	Surgical oncology	1	-	5x2	No	No	-
	Neck re-excision	Surgical oncology	5x2	-	5x1.5	No	No	1
7	Shoulder	Dermatology	1.1x1.1	6	2.3x2.3	No	No	6

Mohs micrographic surgery was used to treat seven patients (Table 2). Patients eight and nine were repeat MMS for previously treated SEDC lesions, and both demonstrated clearance at nine-year and four-year follow-ups. A total of 71.4% (n=5) of cases were cleared with two stages, with two requiring four stages. No cases demonstrated recurrence or metastasis after these surgical interventions, and no cases needed additional adjuvant radiation or chemotherapy.

**Table 2:** Details of Mohs micrographic surgery. *Treatment for recurrent lesions previously managed with MMS. MMS: Mohs micrographic surgery

Patient	Location	Pre-operative size (cm)	Mohs stages	Post-operative size (cm)	Adjuvant radiation	Chemotherapy	Follow-up (months)
8*	Nose	1.0x1.0	4	3.0x3.5	No	No	115
9*	Nose	1.0x1.0	2	1.8x1.6	No	No	52
10	Scalp	1.2x1.0	4	2.5x2.4	No	No	5
11	Arm	0.5x0.5	2	4.5x1.0	No	No	6
12	Forehead	0.9x0.7	2	1.8x1.6	No	No	2
13	Forehead	1.1x0.6	2	1.8x1.8	No	No	135
14	Chest	1.5x1.0	2	2.5x1.5	No	No	113

There was only one case that was originally managed with both MMS and WLE. Initial pre-operative size was 0.9x0.8 cm, which was considered cleared after four stages of MMS with a post-operative surgical defect of 1.6x1.2 cm. The final stage was later determined to have positive margins, for which the patient underwent WLE three months later. The patient received no additional adjuvant radiation or chemotherapy afterward and had no evidence of recurrence six months post-operatively.

Recurrence and metastasis analysis

Of the 27 cases, six had cutaneous lesions that recurred with features concerning for squamoid eccrine ductal carcinomas on repeat biopsies. Of the six cases, one was a pathology consult with limited data, so it was excluded from further analysis.

The average time to recurrence was 19 months, with a range of three to 39 months. Four cases were initially managed with Mohs micrographic surgery. Upon recurrence, two cases were managed with wide local excision, with no evidence of recurrence or metastasis at respective one-year and 11-year follow-ups (patients one and four in Table 1). The other two cases underwent repeat MMS with no evidence of recurrence or metastasis at nine- and four-year follow-up visits (patients eight and nine in Table 2).

One case, originally managed with WLE for a 5 cm “nodular mass” on the shoulder, experienced regional recurrence and metastasis to the forehead three months post-excision. He underwent additional WLE at both sites with adjuvant definitive radiotherapy. Despite these interventions, the disease progressed with recurrence of the forehead lesion, metastasis to a regional lymph node, and the development of a non-healing wound on the shoulder measuring 10.3x5.1x0.3 cm. The patient was started on cisplatin with concurrent radiation, which initially showed a positive response, but the shoulder lesion recurred after eight months. He was then briefly started on cemiplimab before quickly transitioning to carboplatin, paclitaxel, and cetuximab. The patient eventually developed another distant nodule that was concerning for additional metastasis, though the patient denied a biopsy. Ultimately, the patient died from complications related to the shoulder wound, 39 months after the initial biopsy. Of note, his course was complicated by a history of HIV and social stressors that caused frequent lapses in follow-up care.

Histopathologic features

**Figure 2:**
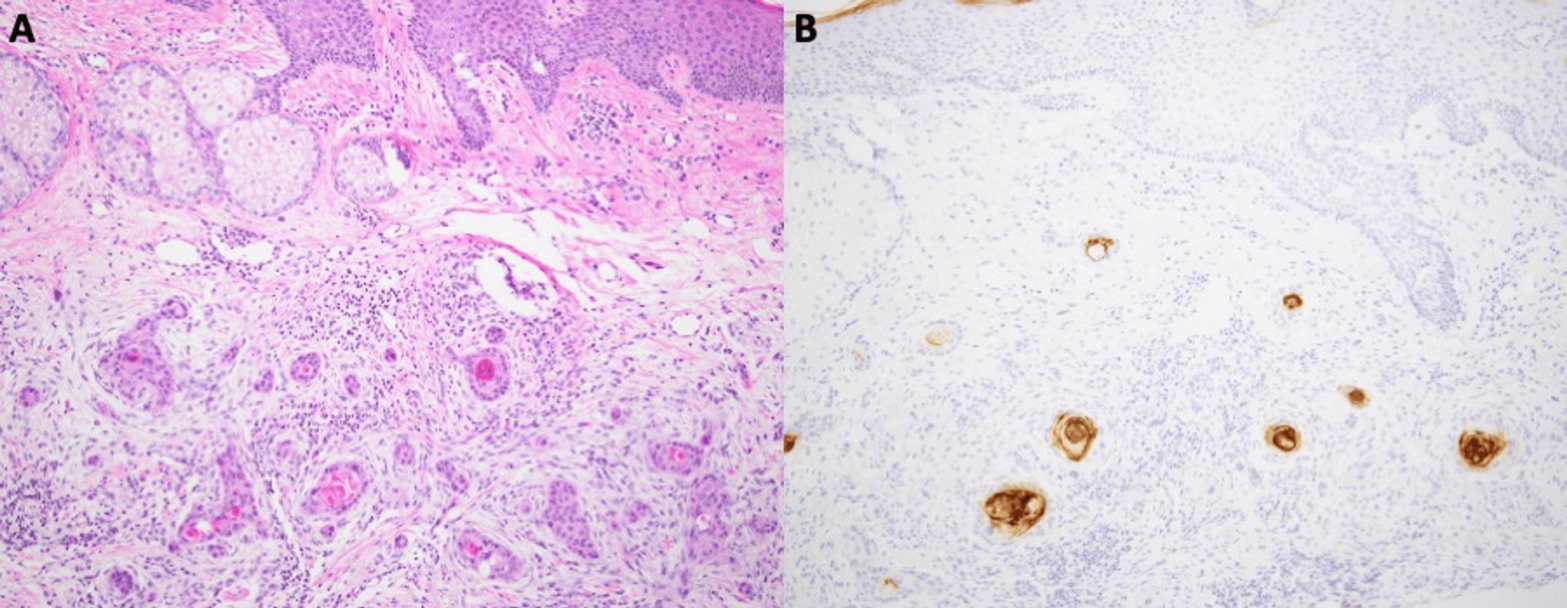
High magnification images (100x) show histologic and immunohistochemical features of SEDC with infiltrative nests and cords of tumor cells in a desmoplastic stroma (H&E stain) (A) with focal ductal differentiation highlighted by CEA (B). SEDC: squamoid eccrine ductal carcinoma; CEA: carcinoembryonic antigen

Eight cases were available for secondary histologic examination. Of these cases, six were shave biopsies, and the remaining two were excisions. One of the excisional cases examined was an excision of a previously biopsied site. For this reason, it was excluded from the remaining analysis for a total of seven unique patients. The most common clinical impression prior to biopsy was basal cell carcinoma and/or squamous cell carcinoma, except for patients undergoing excision. Histologically, all cases exhibited a desmoplastic stromal response with focal tubule differentiation and tumor cells that formed nests and/or cords (Figure 2, panel A). Additionally, 100% cases (n=7) showed some level of solar elastosis in the adjacent dermis, and some even exhibited actinic keratosis or Bowen’s/squamous cell carcinoma in situ disease in the adjacent epidermis (43%, n=3). Most cases (86%, n=6) had associated inflammation comprised of lymphocytes and histiocytes with few plasma cells. Many cases also had features of irritation, such as dermal scar (86%, n=6), overlying serum crust (57%, n=4), or epidermal ulceration (28%, n=2). Necrosis was present in three cases (43%) and tended to occur in the center of tumor nests or within the lumen of ducts. A clear connection to the epidermis was noted in most (71%, n=5) but not all cases. For both cases (n=2, 100%) with excisional tissue extending to the subcutis, subcutaneous extension was noted. Few rare histologic patterns were observed in this cohort, including acantholysis (14%, n=1) and ductal lumen calcifications (14%, n=1). For the case in which the shave and excision were both available for examination, the histologic features were similar; however, the ductal differentiation was not as prominent on the shave as it was in the excision, so the diagnosis prior to excision was not definitive.

## Discussion

In this study, the authors identified 27 cases of squamoid eccrine ductal carcinoma, representing the second largest series of patients with SEDC reported. Clinically, SEDC usually presented less than 2 cm in size as a papule, nodule, or plaque (48.1%, n=13) (Figure 1, panels A-C). One case described an initial mass 5 cm in diameter; however, this may represent the progression of advanced disease. SEDC also demonstrated a predilection for sun-exposed areas, with the head and neck being the most common sites.

Histologically, SEDC shows both squamous and eccrine differentiation [1]. However, the degree of differentiation for these components varies between cases. Additionally, within the same tumor, the proportion of the squamous component is usually higher in the superficial aspect of the lesion. Classically, these tumors exhibit an infiltrative growth pattern that extends to the deep dermis or subcutaneous adipose tissue, and they frequently show a connection to the overlying dermis. SEDCs are typically located in areas of sun damage, and the tumor may appear to arise in a background of actinic keratosis or even Bowen’s/squamous cell carcinoma in situ disease. Their location and previously characterized mutations support an ultraviolet (UV)-related oncogenesis [12,13].

Immunophenotypically, these tumors express both eccrine/adnexal and squamous markers. Typically, there is more squamous expression superficially, and as the tumor extends deeper into the dermis/subcutis, it gains more eccrine markers [1,14]. Based on our institution’s experience, carcinoembryonic antigen (CEA) and epithelial membrane antigen (EMA) were the most useful markers used to confirm eccrine/ductal differentiation (Figure 2, panel B). Because of the morphologic characteristics seen in cells with squamous differentiation and the immunophenotypic overlap with adnexal tissues, no set of immunohistochemical stains will definitively differentiate this tumor from its mimics, so each stain should be integrated with the tumor’s histologic appearance and the patient’s clinical presentation [15,16].

Diagnostically, these tumors pose several challenges, the first of which is due to sampling. As mentioned above, the degree of eccrine differentiation can be extraordinarily heterogeneous throughout the tumor, and superficially, the eccrine component may not be readily apparent. For this reason, superficial biopsies may be indistinguishable from squamous cell carcinoma. In addition to the diagnostic challenges related to sampling, other sweat duct and adnexal malignancies, such as eccrine carcinoma, porocarcinoma, and microcystic adnexal carcinoma, can exhibit histologic features that closely mimic SEDC. However, SEDC often displays more cytologic atypia than microcystic adnexal carcinoma.

While no definitive treatment recommendations exist, wide local excision and Mohs micrographic surgery are the preferred methods of treatment. In this study, the review found an overall recurrence rate of 18.5% (n=5), which aligns with previously reported recurrence rates from 18% to 25% [1,8]. While the majority of these cases were originally managed with MMS, of the seven cases that were treated with MMS at our institution, none demonstrated recurrence or metastasis. This aligns with previously reported recurrence rates of less than 5%, which are thought to be due to better margin control achieved with MMS [6,17,18]. Given our results, MMS may be the preferred surgical modality for treating SEDC due to its potential for better margin control and a decreased rate of recurrence and metastasis; however, comparative studies with WLE are still needed.

Of the nine cases managed with WLE, one case (14.3%) demonstrated recurrence, corresponding to the previously described recurrence rate of 10-70% for lesions managed with conventional surgical excisions [17]. This case was also complicated with metastatic disease to a regional lymph node and distant cutaneous sites despite repeat excisions, chemotherapy, and radiation serving as additional treatment modalities. Ultimately, the patient expired from complications related to the progression of the disease. While barriers to regular follow-up and the patient’s underlying immunosuppression may have played roles in this case, one review of 30 cases reported a metastatic potential of SEDC of 13% (n=3) [1].

The role of adjuvant radiotherapy and chemotherapy has not been well explored in the literature, but both may play roles in decreasing rates of recurrence. In this study, a radiation therapy treatment plan of 6000 centigrays in 30 fractions was used in two cases. Of these, one case had no evidence of recurrence at one-year follow-up. The second case (patient 3), however, ultimately expired from complications of his SEDC disease. Of the 27 cases, only three received chemotherapy. Of these, two cases received either ipilimumab/nivolumab or ipilimumab for concurrent management of melanoma. Consequently, both demonstrated no recurrence at 11-year and one-year follow-ups, respectively. The third case that received chemotherapy as additional SEDC treatment involves patient 3, who received a range of chemotherapeutic agents for his metastatic disease. Overall, given the limited number of cases in this study, the potential benefits of radiation and chemotherapy as adjuvant treatment modalities for SEDC remain unclear.

This study is limited by its retrospective nature and the relatively small number of cases that were available for review. These constraints affect the generalizability of our findings but given the rarity of this neoplasm and the limited number of published cases, this study offers meaningful insight into the clinical behavior, treatment approaches, and outcomes of SEDC. Future studies should aim to continue exploring the clinical features of squamoid eccrine ductal carcinoma, extrapolate the outcome differences between WLE and MMS, and explore if there are any potential benefits of additional treatment modalities.

## Conclusions

In summary, this study presents the second largest review of SEDC cases and discusses the clinical course and histopathologic features of this rare cutaneous neoplasm. Overall, these tumors demonstrated a predisposition for the head and neck and histologically revealed both squamous and eccrine differentiation. They pose a diagnostic challenge as superficial biopsies may not capture deeper activity of the tumors. This study aimed to highlight the importance of recognizing this limitation, given its potential to increase the local recurrence rate, facilitate widespread dissemination, and promote metastasis. Furthermore, although no definitive guidelines currently exist, MMS may be the preferred treatment option due to its lower rates of recurrence.
